# Survival Impact of Residual Cancer Cells in Intraoperative Peritoneal Washes following Radical Hysterectomy for Cervical Cancer

**DOI:** 10.3390/jcm11092659

**Published:** 2022-05-09

**Authors:** Jong Mi Kim, Gun Oh Chong, Nora Jee-Young Park, Yeong Eun Choi, Juhun Lee, Yoon Hee Lee, Dae Gy Hong, Ji Young Park

**Affiliations:** 1 Department of Obstetrics and Gynecology, School of Medicine, Kyungpook National University, Daegu 41944, Korea; jjong9uu@gmail.com (J.M.K.); obgy.ye@gmail.com (Y.E.C.); gyjhlee@knu.ac.kr (J.L.); yhlee1017@knu.ac.kr (Y.H.L.); dghong@knu.ac.kr (D.G.H.); 2 Department of Obstetrics and Gynecology, Kyungpook National University Chilgok Hospital, Daegu 41404, Korea; 3 Clinical Omics Research Center, School of Medicine, Kyungpook National University, Daegu 41944, Korea; 4 Department of Pathology, School of Medicine, Kyungpook National University, Daegu 41944, Korea; jyparkmd@knu.ac.kr

**Keywords:** cervical cancer, residual cancer cells, peritoneal washes, radical hysterectomy, prognosis

## Abstract

Objective: Residual cancer cells (RCCs) contribute to cancer recurrence either because of tumor spillage or undetectable pre-existing micrometastatic tumor clones. We hypothesized that the pathologic evaluation of intraoperative peritoneal washes may reveal RCCs. The aim of this study was to evaluate the survival impact of RCCs identified in intraoperative peritoneal washes and their correlation with clinicopathologic parameters following radical hysterectomy for cervical cancer. Methods: A total of 229 patients with cervical cancer who underwent radical hysterectomy with pelvic and/or paraaortic lymphadenectomy were included. The intraoperative peritoneal washes after surgery were filtered through a strainer and the presence of tumor cells in the residual aspirate was determined. Univariate and multivariate analyses of clinicopathological parameters were performed to identify predictors of recurrence. Results: RCCs in intraoperative peritoneal washes were identified in 19 patients (8.3%). Multivariate analysis revealed that deep stromal invasion (hazard ratio [HR], 13.32; 95% confidence interval [CI], 1.81–98.27; *p* = 0.0111), lymph node metastasis (HR, 2.00; 95% CI, 1.01–3.99; *p* = 0.0482), and neoadjuvant chemotherapy (HR, 2.34; 95% CI, 1.89–4.61; *p* = 0.0139) were associated with tumor recurrence. However, the presence of RCCs was not associated with tumor recurrence (HR, 2.60; 95% CI, 0.74–9.11; *p* = 0.1352). Multiple logistic regression analysis revealed that RCCs were associated with neoadjuvant chemotherapy (odds ratio [OR], 0.22; 95% CI, 0.05–0.99; *p* = 0.0488) and large tumor size (OR, 4.16; 95% CI, 0.77–22.48; *p* = 0.0981). Conclusions: Although the presence of RCCs in intraoperative peritoneal washes do not significantly impact survival outcomes, there was a tendency of inferior survival outcomes in patients with RCCs. RCCs were associated with neoadjuvant chemotherapy and large tumor size.

## 1. Introduction

Radical hysterectomy (RH) is a well-established treatment modality for early-stage cervical cancer. Over the past several decades, minimally invasive surgery (MIS), such as laparoscopy and robot-assisted surgery, has gained widespread acceptance as a standard treatment approach for early-stage cervical cancer, primarily because of better surgical outcomes than open surgery [1,2]. However, the laparoscopic approach to cervical cancer (LACC) trial, a randomized, open-label, noninferiority study that compared minimally invasive RH with open RH, found that MIS was associated with a higher risk of recurrence and death compared with open surgery [3]. The potential reasons for inferior oncologic outcomes following MIS include the routine use of a uterine manipulator, the effect of the insufflation gas (CO_2_) on tumor-cell growth, and an increase in tumor spillage propensity that is due to spread [3]. Moreover, a recent European multicenter, retrospective study reported that the use of uterine manipulator during MIS was associated with worse survival outcomes [4].

During MIS, pneumo-peritoneum may contribute to tumor dissemination, possibly through inhibition of the local peritoneal immune response by CO_2_ or solely the fluctuation in pressure [5,6]. Exposure of tumor cells during intraperitoneal colpotomy to circulating CO_2_ may result in tumor spillage into the peritoneal cavity, especially in MIS for the treatment of cervical cancer. Residual cancer cells (RCCs) from intraoperative peritoneal washes may contribute to cancer recurrence because of tumor spillage or undetectable pre-existing micrometastatic tumor clones [7]. There are two ways how RCCs contribute to cancer recurrence. The first one is ‘synchronous cancer metastasis’. In this way, cancer recurs from microscopic tumors, which cannot be detected via image workup but existed even before the surgery. The second one is ‘metachronous cancer metastasis’. In this way, the cancer cells spread iatrogenicly into the abdominal cavity through intra-operative procedures, which leads to recurrence of the cancer after surgery [7]. Wei et al. reported that the detection of RCCs in intraoperative peritoneal washes of bladder cancer patients undergoing radical cystectomy was associated with tumor aggressiveness and metastatic potential [7]. These characteristics of RCCs and the potential for cancer recurrence are likely to be among the factors that directly contribute to the inferior outcome of MIS. Therefore, we hypothesize that the presence of tumor cells in intraoperative peritoneal washes reflects RCCs, which may represent either tumor spillage during RH or undetectable pre-existing micrometastatic tumor clones.

The purpose of this study is to evaluate the survival impact of RCCs in intraoperative peritoneal washes during RH and to confirm the correlation of RCCs with other clinicopathological factors.

## 2. Materials and Methods

### 2.1. Patient Selection and Clinical Follow-Up

From February 2011 to November 2018, 317 patients who underwent RH for treatment of early-stage and locally advanced cervical cancer were included. Of these patients, 64 of them discarded the samples before analysis of the peritoneal wash obtained after surgery, so the analysis could not be performed. As a result, a total of 253 patients underwent analysis for detecting RCCs in intraoperative peritoneal washes. Six patients who underwent concurrent chemotherapy or preoperative radiotherapy before surgery were excluded and 18 patients with short-term follow-ups, within a period of less than 1 year, were excluded. Finally, a total of 229 remaining patients were analyzed. A uterine manipulator and intraperitoneal colpotomy were used in all cases of MIS RH. Retrospective data collection and analysis were approved by the Institutional Review Board of Kyungpook National University Chilgok Hospital (KNUCH 2020-03-011). The need for informed consent was waived because of the retrospective nature of the study. The patients were clinically staged according to the 2009 International Federation of Gynecologic Obstetrics (FIGO) staging system [8]. The protocol for cancer staging included a pelvic examination under general anesthesia, conization, magnetic resonance imaging of the pelvis, and positron emission tomography/computed tomography.

Clinical follow-ups were performed every 3 months for 2 years, every 6 months after 2 years for up to 5 years, and annually thereafter. Recurrence was confirmed as biopsy-proven or documented disease progression by serial imaging.

### 2.2. Histopathological Evaluation of RCCs

**Figure 1:**
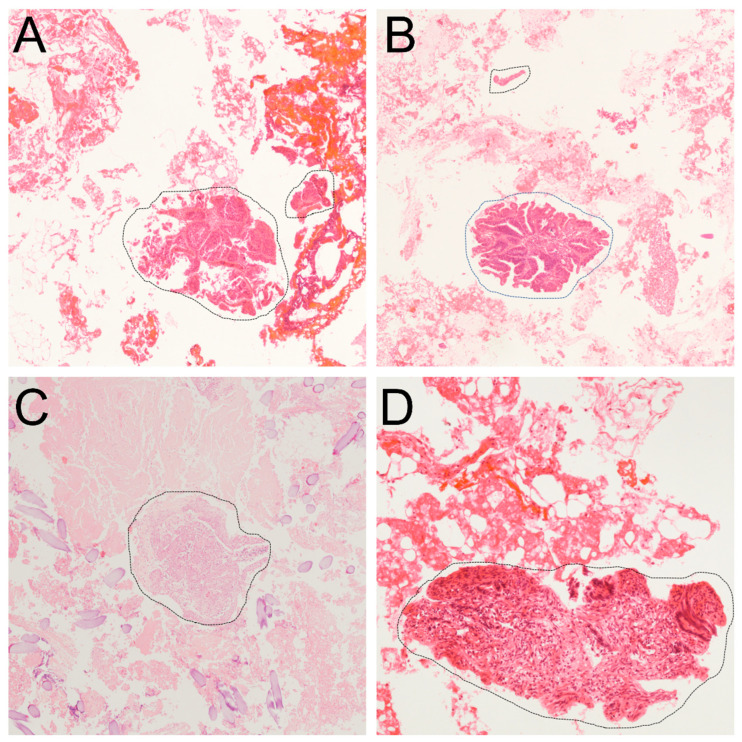
Representative histopathologic features of intraoperative peritoneal washings (**A**–**D**). The collected tissue materials from suction samples exhibited randomly or artificially cut tumor-cell fragments (indicated by black dotted line) within a variable number of mixtures consisting of blood clots, fibrofatty tissue, acute inflammatory cells, acellular proteinaceous materials, and foreign materials, such as gauze or thread. Each image was obtained from tissues of different patients. ((**A**–**D**), hematoxylin and eosin stain; original magnification, (**A**–**C**), ×40; (**D**), ×100).

To identify RCCs from the peritoneal washes, almost all aspirated materials were collected during surgery and filtered through a mesh with a strainer and gauze (Supplementary Figure S1). The aspirated materials were transferred to a 50 mL conical vial and mixed with 10 mL of phosphate buffer saline (PBS). The vial was centrifuged for 10 min at 2500 rpm and the supernatant was discarded. The cell sediment was carefully transferred onto a filter paper and wrapped gently, placed into a cassette, and processed as any other biopsy specimen [9]. Each paraffin-embedded cell block section of 4 μm thickness was stained with hematoxylin and eosin (HE, Figure 1).

For each case, all available specimens were independently reviewed for the detection of RCCs by two pathologists (N.J.P and J.Y.P) in a blinded manner without having information about the clinicopathological data and outcomes. Cases with discrepant results were repeatedly reviewed until a consensus was reached. The pathological parameters included tumor size, FIGO stage, histological subtype, the deepest depth of tumor invasion, and the cervical full-thickness of the area as it applies to evaluating deep stromal invasion (DSI), lymphovascular invasion (LVI), parametrial invasion, and lymph node metastasis.

### 2.3. Statistical Analysis

The time to event was calculated as the time interval from the date of diagnosis to the date of the first evidence of disease recurrence by clinical or imaging examination. The differences between subsets were evaluated by a Student’s *t*-test or Mann–Whitney test, and differences between proportions were compared with the chi-square test. Survival curves for the prognostic factors were estimated using the Kaplan–Meier method and differences between subgroups were compared using the log-rank test. A univariate Cox proportional hazards model was used to determine the hazard ratios of prognostic factors for disease-free survival (DFS) and overall survival (OS). A forward, stepwise multivariate Cox proportional hazards model was used to assess the potential independent effects of prognostic factors for DFS and OS. An estimated hazard ratio (HR) with a 95% confidence interval (95% CI) was calculated. A multiple logistic regression model was used to evaluate the correlation between clinicopathologic parameters and RCCs, and an estimated odds ratio (OR) with a 95% CI was presented. The MedCalc^®^ statistical package (version 12.3.0.0, MedCalc Software, Mariakerke, Belgium) was used for statistical analyses. A *p*-value of less than 0.05 was considered statistically significant. Marginal significance was defined as a *p*-value that ranged from 0.05 to 0.10 [10].

## 3. Results

### 3.1. Clinicopathologic Characteristics

The clinicopathological characteristics of the study participants are listed in Table 1. The predominant FIGO stage was IB1 (*n* = 162 [70.7%]), followed by IB2 (*n* = 33 [14.4%]), IIB (*n* = 22 [9.6%]), IIA1 (*n* = 8 [3.5%]), and IIA2 (*n* = 4 [1.7%]). The histological cervical cancer types were as follows: squamous cell carcinoma (*n* = 153 [66.8%]) and adenocarcinoma/adenosqaumous carcinoma (*n* = 76 [33.2%]). MIS was performed in 208 patients (90.8%), whereas open surgery was performed in 21 patients (9.2%). Nineteen patients (8.3%) had RCCs in intraperitoneal washes.

### 3.2. Treatment Outcomes

Thirty-eight patients (16.6%) received concurrent adjuvant chemoradiotherapy for high-risk factors including lymph node metastasis, parametrial invasion, or positive resection margin, and 23 patients (10%) received adjuvant radiotherapy for intermediate-risk factors. Forty-nine patients (21.4%) received adjuvant chemotherapy for high- or intermediate-risk factors.

After a median follow-up of 59 months (6–123 months), 35 patients (15.3%) had a recurrence and 13 patients (5.7%) died from disease progression.

### 3.3. Survival Analyses

From the univariate analysis, stage (HR, 2.67; 95% CI, 1.26–5.67; *p* = 0.0105), histology (HR, 2.12; 95% CI, 1.04–4.32; *p* = 0.0381), tumor size (HR, 2.14; 95% CI, 1.10–4.16; *p* = 0.0252), DSI (HR, 3.73; 95% CI, 1.81–7.68; *p* = 0.0003), parametrial invasion (HR, 3.10; 95% CI, 1.26–7.65; *p* = 0.0140), pretreatment LEEP (HR, 0.33; 95% CI, 0.16–0.70; *p* = 0.0036), neoadjuvant chemotherapy (HR, 2.67; 95% CI, 1.21–5.91; *p* = 0.0150), and lymph node metastasis (HR, 4.25; 95% CI, 1.75–10.30; *p* = 0.0014) were significant prognostic indicators of DFS. The forward stepwise multivariate Cox proportional hazards model revealed that deep stromal invasion (HR, 13.32; 95% CI, 1.81–98.27; *p* = 0.0111), neoadjuvant chemotherapy (HR, 2.34; 95% CI, 1.89–4.61; *p* = 0.0139), and lymph node metastasis (HR, 2.00; 95% CI, 1.01–3.99; *p* = 0.0482) were significant prognostic factors for DFS (Table 2). From the multivariate analysis, age (HR, 5.37; 95% CI, 1.77–16.28; *p* = 0.0030) and tumor size (HR, 9.58; 95% CI, 1.20–76.38; *p* = 0.0329 were significantly associated with OS (Table 3).

**Figure 2:**
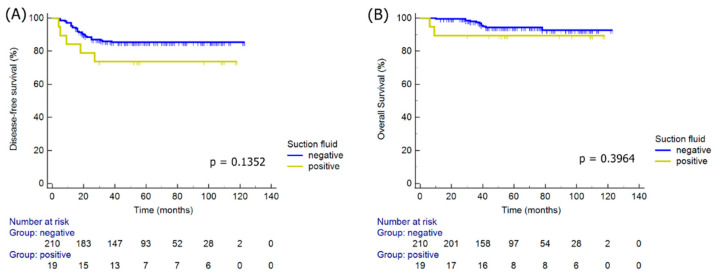
Kaplan–Meier survival plots for disease-free survival (**A**) and overall survival (**B**) based on residual cancer cells status.

The presence of RCCs in the intraperitoneal washes was not an independent prognostic factor for DFS (HR, 2.60; 95% CI, 0.74–9.11; *p* = 0.1352) or OS (HR, 2.30; 95% CI, 0.34–15.81; *p* = 0.3964). Moreover, survival outcomes were not significantly different based on positive RCCs in the intraperitoneal washes based on the Kaplan–Meier survival plots. Although no statistical difference in DFS was evident according to the presence of RCCs, an inferior DFS was not identified in the positive RCCs groups compared with the RCC-negative group (Figure 2).

### 3.4. Clinicopathologic Variables Associated with Positive RCC

We analyzed the clinicopathologic variables associated with positive RCCs during RH. Multiple logistic regression analyses indicated that neoadjuvant chemotherapy (OR, 0.22; 95% CI, 0.05–0.99; *p* = 0.0488) was significantly correlated with positive RCCs. Furthermore, large tumor size (OR, 4.16; 95% CI, 0.77–22.48; *p* = 0.0981) and parametrial invasion (OR, 3.28; 95% CI, 0.85–12.63; *p* = 0.0846) were positively correlated with the presence of RCCs with marginal significance. However, MIS was not associated with positive RCCs (OR, 0.72; 95% CI, 0.17–2.96; *p* = 0.6495) (Table 4).

## 4. Discussion

With the development of endoscopic technology, the open surgery has naturally shifted to a less invasive MIS, which provides not only a quick recovery but also a cosmetic satisfaction after the surgery. The clear and magnified surgical field provided by MIS enables more delicate surgery and it may also contribute to surgical education. However, as a recent study revealed, MIS showed inferior oncologic outcomes compared to the open surgery of cervical cancer patients. Therefore, the surgical method tends to return to the open surgery. There have been many studies on the associated factors of the high rate of tumor recurrence and death in MIS. We hypothesized that RCCs could be one of the associated factors and it could be identified in peritoneal washings obtained during surgery. According to this hypothesis, we conducted the current study to evaluate the survival impact of RCCs after surgery in early cervical cancer patients and identified the factors related to RCCs. To the best of our knowledge, this is the first study on the survival impact of RCCs during radical hysterectomy of early cervical cancer.

In this study, we evaluated the survival impact of RCCs in intraoperative peritoneal washes and its correlation with clinicopathologic characteristics following RH in cervical cancer. RCCs in intraoperative peritoneal washes did not impact survival outcomes during RH during the treatment of cervical cancer; however, neoadjuvant chemotherapy was associated with RCCs.

In the LACC trials, the potential reasons for the inferior oncologic outcomes for MIS included the routine use of a uterine manipulator, the effect of the insufflation gas (CO_2_) on tumor-cell growth, and the spread during intraperitoneal colpotomy that may increase the propensity for tumor spillage [3]. In the SUCCOR study, the use of a uterine manipulator during MIS was associated with a worse 4.5-year DFS (82% vs. 93%; HR, 3.48; 95% CI, 1.17–9.48, *p* = 0.028) and 4.5-year OS (88% vs. 96%; *p* = 0.016) [4]. However, Nica et al. demonstrated that the use of an intrauterine manipulator in patients with early cervical cancer who underwent MIS RH was not an independent factor associated with tumor recurrence after adjusting for adverse pathological factors (HR, 0.4; 95% CI, 0.2–1.0; *p* = 0.05) [11]. Kong et al. demonstrated that the rate of disease recurrence was higher in the intracorporeal colpotomy group compared with the vaginal colpotomy group (16% vs. 5%). Among the patients with recurrence in the intracorporeal group, 62% had intraperitoneal spread or carcinomatosis [12]. It was concluded that exposure of cervical cancer to circulating CO_2_ may result in tumor spillage into the peritoneal cavity during intraperitoneal colpotomy [13]. Furthermore, other studies reported intraperitoneal recurrences only from the robot or MIS during RH [13,14]. Klapdor et al. evaluated peritoneal contamination with indocyanine green-stained cervical secretion as a surrogate for potential cervical cancer cell dissemination during colpotomy [15]. Peritoneal contamination was observed in 75% (9/12) of the patients during laparoscopic hysterectomy and uterine manipulator contamination was detected in 60% [15]. In the present study, a uterine manipulator and intraperitoneal colpotomy were used in all cases of MIS RH. Although the presence of RCCs in intraperitoneal washes was not an independent prognostic factor for survival, there was a tendency for inferior survival outcomes in the presence of RCC. In addition, 16 patients (7.7%) who received MIS RH had RCCs in intraoperative peritoneal washes. This indicates that studies should be performed on how to avoid the use of uterine manipulators and the dissemination of cancer cells by ensuring a more effective vaginal closure by standardized methods.

Wei et al. quantified RCC levels as the relative cancer cell fraction and RCCs were detected in approximately half of the pelvic wash specimens collected during or after robotic-assisted radical cystectomy [7]. Moreover, higher levels of RCCs were associated with aggressive variant histology and tumor recurrence [7]. Detection of RCCs in intraoperative peritoneal washes may represent a robust marker for tumor aggressiveness and metastatic potential [7]. Zoli et al. introduced a suction filter into endoscopic endonasal surgery [16]. The use of a filter may facilitate the collection of the largest amount of pathological tissue possible for each surgery [16]. RCCs in intraoperative peritoneal washes, which were filtered through a mesh of strainer and gauze, may yield important clinicopathological information, not only in cervical cancer but also other malignancies.

In the present study, we defined the source of RCCs in peritoneal washes, whether it was from an incidental tumor spillage and/or from previously existing micrometastasis. During cancer progression, peritoneal dissemination requires sequential processes in which malignant cells invade the exposed sub-mesothelial connective tissues and attach to the disrupted mesothelial surface. This results in the specified tissue reactions, such as tumor-promoting inflammation and epithelial-mesenchymal transition [17]. Only truly invasive cancer cells originating from undetectable pre-existing micrometastasis can contribute to these specific occurrences. Upon careful microscopic examination, all samples containing RCCs from peritoneal washes revealed randomly or artificially cut tumor-cell fragments within a variable number of mixtures consisting of blood clots, fibrofatty tissue, acute inflammatory cells, acellular proteinaceous materials, and foreign bodies, such as gauze or thread (Figure 1). These histopathologic features do not provide definitive evidence for interactive tissue reactions, in other words, peritoneal dissemination as a part of true cancer progression among the tumor cells, stromal cells of adjacent connective tissues, mesothelial cells, or inflammatory infiltrates. Thus, the presence of RCCs in intraoperative peritoneal washes may originate from accidental tumor spillage. Although the determination of RCCs from peritoneal washes as incidental tumor contamination remains controversial and the presence of RCC raises concerns regarding inferior outcomes and should not be overlooked.

We demonstrated that the large tumor size was positively correlated with the presence of RCCs. In addition, multiple regression analysis showed that neoadjuvant chemotherapy was negatively correlated with the presence of RCCs. These findings suggest that neoadjuvant chemotherapy may prevent or minimize tumor spillage during MIS RH when a large tumor or incomplete surgical resection is a concern prior to surgery. In recent years, as an alternative treatment for large cervical tumors or locally advanced cervical cancer, neoadjuvant chemotherapy has been performed before RH to reduce large tumor size [18,19]. Although there are reports that neoadjuvant chemotherapy does not affect overall survival, even if the tumor size is reduced [20], it is necessary to further evaluate the clinicopathologic contribution of neoadjuvant chemotherapy to preventing or minimizing tumor-cell spillage during MIS RH and its effect on the delay in local progression or extension of the time to recurrence.

The main limitations to this study include its retrospective nature and the small sample size, which may have contributed to selection bias. In addition, it represents a single-center study; thus, the generalization of our findings is partly limited. Finally, the filtrate of intraoperative peritoneal washes does not represent all RCCs, especially micrometastasis. Despite these limitations, our study offers some unique and significant findings. The incidence and survival impact of RCCs during RH was first described in this study. Moreover, clinicopathological factors that correlate with RCCs were evaluated.

## 5. Conclusions

RCCs in intraoperative peritoneal washes do not impact survival outcomes during RH for the treatment of cervical cancer. Neoadjuvant chemotherapy was associated with RCCs. Although RCCs were not significantly associated with tumor recurrence, the incidence of RCCs during RH was 8.3%. Although no statistical difference in DFS was observed based on the presence of RCCs, there was a tendency of inferior survival outcomes in the RCC-positive group compared with the negative group. Therefore, tumor spillage should be a concern during RH, particularly for intraperitoneal colpotomy.

## Figures and Tables

**Table 1:** Clinicopathologic characteristics of cervical cancer patients with and without recurrence.

Variables	All (*n* = 229)	No Recurrence (*n* = 194)	Recurrence (*n* = 35)	*p* Value
Age (years)	49.99 ± 10.30	50.28 ± 10.14	48.40 ± 11.17	0.3218
FIGO stage (*n*, %)				0.0335
IB1	162 (70.7)	143 (73.7)	19 (54.3)	
IB2	33 (14.4)	25 (12.9)	8 (22.9)	
IIA1	8 (3.5)	5 (2.6)	3 (8.6)	
IIA2	4 (1.7)	2 (1.0)	2 (5.7)	
IIB	22 (9.6)	19 (9.8)	3 (8.6)	
Histology (*n*, %)				0.0361
SCC	153 (66.8)	135 (69.6)	18 (51.4)	
AC/ASC	76 (33.2)	59 (30.4)	17 (48.6)	
Tumor size (cm)	2.23 ± 1.75	2.08 ± 1.74	3.07 ± 1.57	0.0019
Lymphovascular invasion (*n*, %)	81 (35.4)	66 (34.0)	15 (42.9)	0.3153
Deep stromal invasion (*n*, %)	164 (71.6)	130 (67.0)	34 (97.1)	0.0003
Parametrial invasion (*n*, %)	42 (18.3)	31 (16.0)	11 (31.4)	0.0301
Lymph node metastasis (*n*, %)	43 (18.8)	30 (15.5)	13 (37.1)	0.0026
Residual cancer cells (*n*, %)	19 (8.3)	14 (7.2)	5 (14.3)	0.1638
Preoperative LEEP (*n*, %)	60 (26.2)	58 (29.9)	2 (5.7)	0.0028
Neoadjuvant chemotherapy (*n*, %)	57 (24.9)	43 (22.2)	14 (40.0)	0.025
Adjuvant therapy (*n*, %)				0.0026
CCRT	38 (16.6)	26 (13.4)	12 (34.3)	
Chemotherapy	49 (21.4)	42 (21.6)	7 (20.0)	
Radiotherapy	23 (10.0)	17 (8.8)	6 (17.1)	
Surgical approach				0.1606
Minimally invasive surgery	208 (90.8)	174 (89.7)	34 (97.1)	
Open surgery	21 (9.2)	20 (10.3)	1 (2.9)	

AC = adenocarcinoma; ASC = adenosquamous carcinoma; CCRT = concurrent chemoradiotherapy; FIGO = International Federation of Gynecology and Obstetrics; LEEP = loop electrosurgical excision procedure; SCC = squamous cell carcinoma.

**Table 2:** Univariate and multivariate analyses of clinical variables for the prediction of tumor recurrence.

Variables	Univariate Analysis			Multivariate Analysis		
	HR	95% CI	*p*	HR	95% CI	*p*
Age (years) ≤40 vs. >40	2.17	0.87–5.42	0.0980			
FIGO stage ≥IB2 vs. IB1	2.67	1.26–5.67	0.0105			
Histology AC/ASC vs. SCC	2.12	1.04–4.32	0.0381			
Tumor size >2 cm vs. ≤2 cm	2.14	1.10–4.16	0.0252			
Lymphovascular invasion	1.46	0.72–2.93	0.2926			
Deep stromal invasion	3.73	1.81–7.68	0.0003	13.32	1.81–98.27	0.0111
Parametrial invasion	3.10	1.26–7.65	0.0140			
Lymph node metastasis	4.25	1.75–10.30	0.0014	2.00	1.01–3.99	0.0482
Positive residual cancer cell	2.60	0.74–9.11	0.1352			
Preoperative LEEP	0.33	0.16–0.70	0.0036			
Neoadjuvant chemotherapy	2.67	1.21–5.91	0.0150	2.34	1.89–4.61	0.0139
Minimally invasive surgery	2.15	0.68–6.79	0.1929			

AC = adenocarcinoma; ASC = adenosqumous carcinoma; FIGO = International Federation of Gynecology and Obstetrics; LEEP = loop electrosurgical excision procedure; SCC = squamous cell carcinoma.

**Table 3:** Univariate and multivariate analyses of clinical variables for the prediction of death.

Variables	Univariate Analysis			Multivariate Analysis		
	HR	95% CI	*p*	HR	95% CI	*p*
Age (years) ≤40 vs. >40	7.70	1.82–32.59	0.0055	5.37	1.77–16.28	0.0030
FIGO stage ≥IB2 vs. IB1	5.05	1.51–16.84	0.0084			
Histology AC/ASC vs. SCC	1.90	0.59–6.11	0.2786			
Tumor size >2 cm vs. ≤2 cm	5.34	1.80–15.86	0.0025	9.58	1.20–76.38	0.0329
Lymphovascular invasion	1.19	0.38–3.74	0.7647			
Deep stromal invasion	3.01	0.93–9.75	0.0665			
Parametrial invasion	7.77	1.83–32.96	0.0054	2.92	0.95–9.01	0.0623
Lymph node metastasis	6.54	1.60–26.80	0.0090			
Positive residual cancer cell	2.30	0.34–15.81	0.3964			
Preoperative LEEP	0.37	0.11–1.25	0.1092			
Neoadjuvant chemotherapy	4.94	1.39–17.54	0.0134			
Minimally invasive surgery	1.08	0.15–7.79	0.9362			

AC = adenocarcinoma; ASC = adenosqumous carcinoma; FIGO = International Federation of Gynecology and Obstetrics; LEEP = loop electrosurgical excision procedure; SCC = squamous cell carcinoma.

**Table 4:** Clinicopathologic variables associated with positive residual cancer cells.

Variables	OR	95% CI	*p* Value
Age (years) ≤40 vs. >40	0.95	0.21–4.24	0.9427
FIGO stage ≥IB2 vs. IB1	2.46	0.65–9.34	0.1842
Histology AC/ASC vs. SCC	0.44	0.12–1.65	0.2222
Tumor size >2 cm vs. ≤2 cm	4.16	0.77–22.48	0.0981
Lymphovascular invasion	0.32	0.08–1.25	0.1013
Deep stromal invasion	-	-	0.9978
Parametrial invasion	3.28	0.85–12.63	0.0846
Lymph node metastasis	1.38	0.35–5.38	0.6450
Preoperative LEEP	0.90	0.14–5.70	0.9080
Neoadjuvant chemotherapy	0.22	0.05–0.99	0.0488
Minimally invasive surgery	0.72	0.17–2.96	0.6495
Recurrence	1.91	0.44–8.34	0.3875
Death	0.61	0.07–5.57	0.6574

AC = adenocarcinoma; ASC = adenosqumous carcinoma; FIGO = International Federation of Gynecology and Obstetrics; LEEP = loop electrosurgical excision procedure; SCC = squamous cell carcinoma.

## Data Availability

The data presented in this study are available upon request from the corresponding authors.

## Supplementary Materials

The following supporting information can be downloaded at https://www.mdpi.com/article/10.3390/jcm11092659/s1: Supplementary Figure S1: Residual aspirates of intraoperative peritoneal washes during redial hysterectomy.
