# Supplementary material for: Survival Impact of Residual Cancer Cells in Intraoperative Peritoneal Washes following Radical Hysterectomy for Cervical Cancer
Source: J Clin Med. 2022 May 9;11(9):2659. doi: 10.3390/jcm11092659 (PMC9102082; doi:10.3390/jcm11092659)
Supplement: Supplementary file 1 [file jcm-11-02659-s001.zip › jcm-1713046-supplementary.pdf]

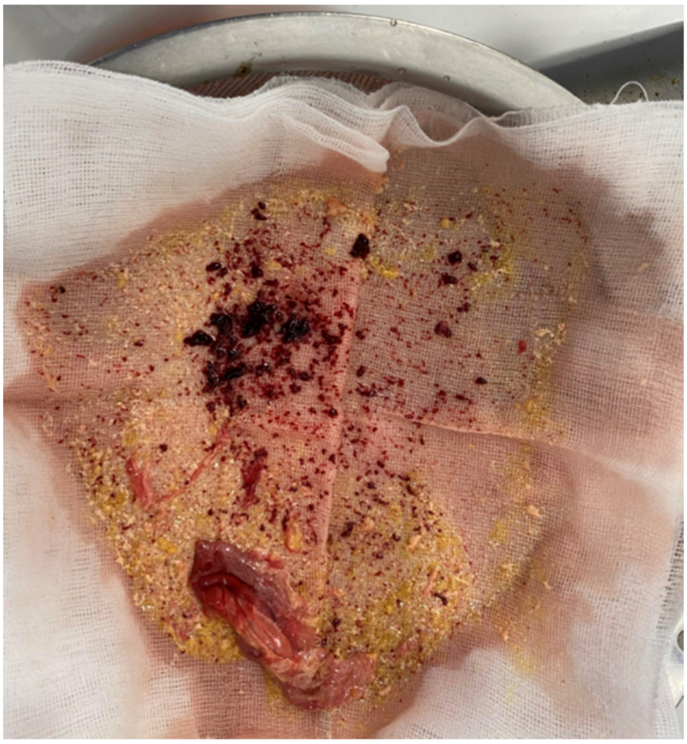

Figure S1. Residual aspirates of intraoperative peritoneal washes during redial hysterectomy.
